# Gene co-expression in the interactome: moving from correlation toward causation via an integrated approach to disease module discovery

**DOI:** 10.1038/s41540-020-00168-0

**Published:** 2021-01-21

**Authors:** Paola Paci, Giulia Fiscon, Federica Conte, Rui-Sheng Wang, Lorenzo Farina, Joseph Loscalzo

**Affiliations:** 1 Department of Computer, Control and Management Engineering, Sapienza University of Rome, Rome, Italy; 2 Institute for Systems Analysis and Computer Science “Antonio Ruberti”, National Research Council, Rome, Italy; 3 Fondazione per la Medicina Personalizzata, Via Goffredo Mameli, 3/1 Genova, Italy; 4 Department of Medicine, Brigham and Women’s Hospital, Harvard Medical School, Boston, MA 02115 USA

**Keywords:** Diseases, Computational biology and bioinformatics

## Abstract

In this study, we integrate the outcomes of co-expression network analysis with the human interactome network to predict novel putative disease genes and modules. We first apply the SWItch Miner (SWIM) methodology, which predicts important (switch) genes within the co-expression network that regulate disease state transitions, then map them to the human protein–protein interaction network (PPI, or interactome) to predict novel disease–disease relationships (i.e., a SWIM-informed diseasome). Although the relevance of switch genes to an observed phenotype has been recently assessed, their performance at the system or network level constitutes a new, potentially fascinating territory yet to be explored. Quantifying the interplay between switch genes and human diseases in the interactome network, we found that switch genes associated with specific disorders are closer to each other than to other nodes in the network, and tend to form localized connected subnetworks. These subnetworks overlap between similar diseases and are situated in different neighborhoods for pathologically distinct phenotypes, consistent with the well-known topological proximity property of disease genes. These findings allow us to demonstrate how SWIM-based correlation network analysis can serve as a useful tool for efficient screening of potentially new disease gene associations. When integrated with an interactome-based network analysis, it not only identifies novel candidate disease genes, but also may offer testable hypotheses by which to elucidate the molecular underpinnings of human disease and reveal commonalities between seemingly unrelated diseases.

## Introduction

The new emerging paradigm of *network medicine* has been dramatically changing the way we define and analyze human diseases. Rather than view a disease as an independent entity, the network medicine approach recognizes the interplay of multiple molecular processes in expressing the pathophenotype^1–3^. By proposing a holistic approach according to which characterizing the behavior of the network as a whole is essential for understanding disease complexity^4^, network medicine sets the stage for exploring disease complexity at the cellular and molecular levels, and for studying the relationships among apparently different pathophenotypes. A key goal of network medicine is to gain a better understanding of how perturbations propagate through the system by identifying and characterizing potential network modules that can be targeted for clinical intervention. Although introduced relatively recently, scientific research in the network medicine field has been growing rapidly as witnessed by the number of evolving methods designed to interrogate disease etiology, model molecular and genetic interactions, identify potential biomarkers, and design therapeutic interventions, including both drug discovery and drug repurposing^1,5–18^.

The two key issues that each network-based algorithm has to address are the identification of the critical entities in the system under investigation (nodes), and the nature of the interactions between these entities (edges). This information depends on the study design, the phenotype under investigation, the biological question of interest, the molecular entities measured, and the type and size of the available datasets. Thus, tools developed within the field of network medicine are highly customized to leverage these biomedical data with respect to the given biological or disease context. Several of these algorithms^5–7^ make use of the human protein–protein interaction (PPI) network, also denoted the human interactome, which is a network of proteins (nodes) in which the edges are the physical and functional interactions occurring between them. Despite the fundamental insights PPI networks provide about the topological features of specific protein interactions within them, their intrinsic immutable nature renders them void of context-specific information (i.e., cell-, tissue-, or disease-specificity). Moreover, the incompleteness of the current interactome and the partial knowledge of the number of genes associated with various diseases make mining disease-specific interactions via the PPI network a very demanding task. To overcome all of these shortcomings, novel in silico strategies are necessary to overlay the interactome with this additional, important biomedical information.

Gene expression networks (GENs) are context-specific by definition, as they directly leverage phenotype-specific gene expression data in network construction by calculating correlations between the expression profiles of each gene pair. The basic premise of this exercise is that, even though correlation is not causation, co-expressed genes are functionally coordinated in response to an external stimulus, implying that they may be part of the same complexes or pathways, and may influence each other or may be influenced by the same underlying mechanism(s). For example, SWItch Miner (SWIM)^19^ is a new, promising methodology that considers differentially expressed genes within the co-expression network framework in order to predict important genes affected by a disease of interest, and combines this information with a structured network of correlated patterns. Considering the topological properties of the nodes and assessing their functional roles according to their ability to convey information within and between modules in the network, SWIM identifies a small pool of genes (known as *switch genes*) that are associated with intriguing patterns of molecular co-abundance and play a crucial role in the observed phenotype (transitions). The phenotype-specific applications of SWIM are broad and include the identification of switch genes in both complex diseases and cancers^19–23^, and as well as in grapevine berry maturation (*Vitis vinifera*)^24^.

In cancer research, SWIM network analysis has been gainfully applied to a large panel of TCGA (The Cancer Genome Atlas) cancer datasets^25^ in order to characterize disease etiologies and identify potential therapeutic targets^19^. SWIM has also been used to investigate glioblastoma multiforme and to uncover new insights into the molecular mechanism determining the stem-like phenotype of glioblastoma cells^20^. [Stem-like cells determine tumor aggressiveness by sustaining tumor growth and causing relapse and metastasis by their resistance to conventional cancer therapies^26^.] In particular, the role of FOSL1 was explored and found to be a repressor of a core of four master neurodevelopmental transcription factors whose induction is sufficient to reprogram fully differentiated glioblastoma cells into stem-like cells^27^. This result could have a significant impact on personalized healthcare, since promoting differentiation and restraining tumor growth may support rational, personalized planning of disease prevention or treatment.

Recently, SWIM methodology has been successfully applied to chronic obstructive pulmonary disease (COPD)^22^, a severe lung disease characterized by progressive and incompletely reversible airflow obstruction. COPD is a heterogeneous and complex syndrome influenced by both genetic and environmental determinants, and is one of the main causes of morbidity and mortality worldwide. COPD switch genes appear to form localized connected subnetworks displaying an intriguingly common pattern of upregulation in COPD cases compared with controls. A more sophisticated analysis revealed that they were not only topologically related, but also functionally relevant to the observed phenotype as witnessed by their enrichment in the regulation of inflammatory and immune responses. The results obtained in COPD were compared with those obtained in the acute respiratory distress syndrome (ARDS), another severe lung disease with an inflammatory component. Interestingly, ARDS switch genes were different from COPD switch genes, but the major pathways affected in the two diseases were similar, emphasizing that different diseases often have common underlying mechanisms and share intermediate endophenotypes (convergent phenotypes)^6,28^. Moreover, the two lists of switch genes, when mapped to the human interactome, appear to form non-overlapping modules and to be situated in different network neighborhoods. This observation demonstrates that even though different diseases can share similar endophenotypes, the molecular network determinants responsible for them are disease-specific. This observation is also fully consistent with the fundamental principles of network medicine, where disease proteins are assumed not to be randomly scattered, but agglomerate in specific regions of the molecular interactome, suggesting the existence of specific disease network modules for each disease.

Inspired by the results obtained by SWIM network analysis of cancers and COPD, here we investigated three other complex diseases for a more generalizable understanding of the highly interconnected nature of human diseases. Specifically, two cardiac disorders, ischemic and non-ischemic cardiomyopathy, and one neurodegenerative disorder, Alzheimer’s disease (AD), were analyzed. These new results, together with the previously obtained analyses from the application of SWIM to ten tumor types and COPD, were mapped to the human interactome in order to overlay the PPI network with disease information derived from SWIM-based disease correlation networks.

Our goal is to assess the utility of SWIM network analysis in classifying several different disorders and in understanding their complex interconnections in the human interactome. In particular, through the construction of a SWIM-informed human disease network (SHDN) by analogy with ref. ^5^, we test whether or not switch genes of a specific disease tend to localize in a critical module in the interactome that is functionally relevant to the observed phenotype.

## Results

### Workflow of the analysis

**Fig. 1:**
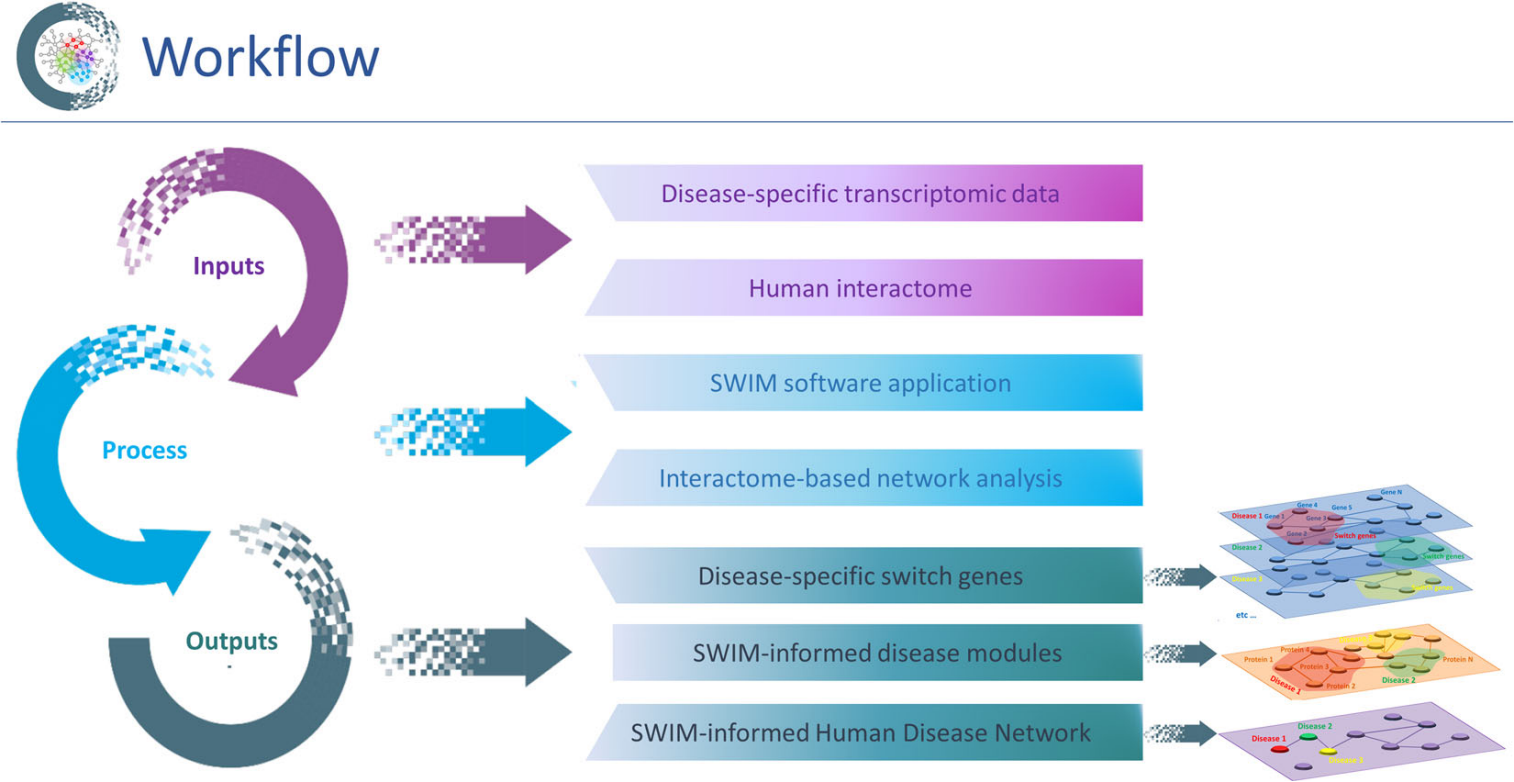
Workflow of the analysis. Our analysis takes as input the transcriptomic data of the 14 diseases of interest and the human interactome network. The analysis begins with the application of the SWIM algorithm to construct the disease-specific Gene Expression Networks (GENs), and, hence, to identify the disease-specific switch genes. Next, by mapping the disease-specific switch genes to the human interactome, the analysis next proceeds with an interactome-based network analysis, which reveals the SWIM-informed disease modules and the SWIM-informed Human Disease Network (SHDN), in which nodes are now diseases, and a link occurs if the corresponding switch gene modules were found to overlap.

In this study, we combined the topological properties of the human interactome with disease information derived from SWIM-based correlation network analysis. The baseline networks of our analysis are SWIM-based GENs and the outcome network is an SHDN, by analogy with a previous study^5^. The workflow of our study design is depicted in Fig. 1.

### Identification of disease-specific switch genes

The SWIM algorithm was applied to a specific group of diseases of interest to build disease-specific GENs (Supplementary Data 1) and extract a list of switch genes for each disease through an accurate topological analysis (Supplementary Table 2). The analyzed human diseases were:

(i) ten tumor types (i.e., BLCA, BRCA, CHOL, COAD, HNSC, KIRP, LUAD, LUSC, PRAD, and UCEC) available from TCGA, whose corresponding lists of switch genes were retrieved from our previous study^11^;
(ii) one pulmonary disease (COPD), whose corresponding list of switch genes was retrieved from our previous study^14^;
(iii) ischemic cardiomyopathy (IC), whose list of switch genes was obtained by applying SWIM correlation network analysis to RNA-sequencing data from ischemic human failing versus non-failing control hearts; and
(iv) non-ischemic cardiomyopathy (NIC), whose list of switch genes was obtained by applying SWIM correlation network analysis to RNA-sequencing data from non-ischemic human failing versus non-failing control hearts;
(v) AD, whose list of switch genes was obtained by applying SWIM correlation network analysis to microarray expression data related to AD patients versus controls.

### Identification of SWIM-informed disease modules

**Fig. 2:**
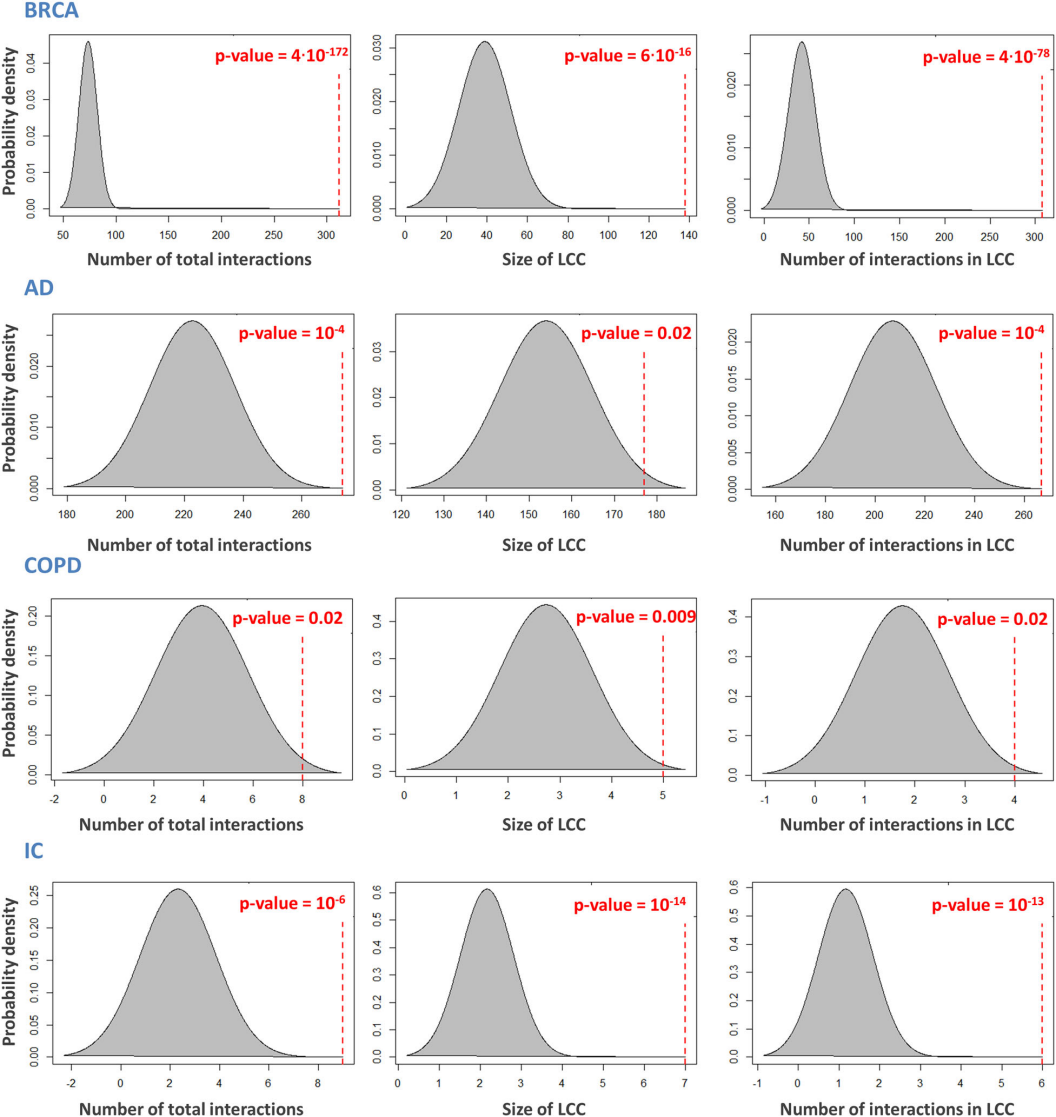
Examples of SWIM-informed disease module. From left to right: Distribution of the number of total interactions, the size of the largest connected component (LCC), and the number of edges in the LCC in the subgraph induced by a randomly selected gene set of the same size and degree distribution as the original disease list of the switch genes in the human interactome. Dashed red lines correspond to the observed values of each metric computed for the list of switch genes mapped to the interactome. All *p*-values were calculated by using a one-tailed *z* test.

Actually, members (nodes, proteins, or genes) of a network module are more functionally and topologically related to each other than to other nodes in the network. Thus, the lists of switch genes for the 14 analyzed diseases were mapped onto the human interactome to investigate whether or not they tend to agglomerate in local neighborhoods and constitute statistically significant disease-specific modules. To do so, for each disease, the corresponding switch genes subnetwork was extracted and the following three metrics were computed: (i) the total number of interactions (edges); (ii) the size of the largest connected component (LCC); and (iii) the number of edges in the LCC. In order to complement these metrics with a measure of statistical significance, we computed *module significance*, which measures the probability that a given list of switch genes is localized within a certain network neighborhood more than expected by chance. For each disease, we randomly selected groups of proteins of the same size and degree distribution as the original list of switch genes in the human interactome. We then extracted the corresponding subnetwork and we computed: (i) the total number of its interactions; (ii) the size of the LCC; and (iii) the number of edges in the LCC. This procedure was repeated 1000 times. [As reported in the majority of state-of-the-art approaches^29–37^, 1000 permutations is commonly used for estimating the power of a randomization test, showing it that can be considered as a reasonable number of permutations for a test at the 5% level of significance.] Finally, we derived three distributions for all three metrics corresponding to the subgraph induced by the random gene set. The three metrics calculated for the original list of switch genes were *z*-score-normalized with respect to the corresponding reference random distribution. Subsequently, the *p*-value for the given *z* statistic was calculated. We found that all of the analyzed sets of switch genes form statistically significant modules (i.e., all three metrics were statistically significant) in the human interactome that are disease-specific (Fig. 2 and Supplementary Data 3).

### Overlap estimation of SWIM-informed disease modules

In order to evaluate the extent to which two disease-specific modules (A, B) of switch genes are in the immediate vicinity of each other in the human interactome, we leveraged the *module separation* parameter defined in Eq. (1) (cf. “Methods”) that measures the separation or overlap of two modules^6^, and we applied a degree-preserving randomization procedure to assess the statistical significance of each separation value. By analyzing the topological structure of the identified SWIM-informed disease modules, we found that three topologically different situations came to light:

1. two given modules overlap more than expected by chance (i.e., *s* < 0 and *p*-value < 0.05), hereafter denoted *cognate modules*;
2. two given modules separate more than expected by chance (i.e., *s* > 0 and *p*-value < 0.05), hereafter denoted *non-cognate modules*; and
3. there is insufficient evidence to support the hypothesis that two given modules overlap or separate more than expected by chance (i.e., *p*-value > 0.05), hereafter denoted *modules of uncertain overlap*.

**Fig. 3:**
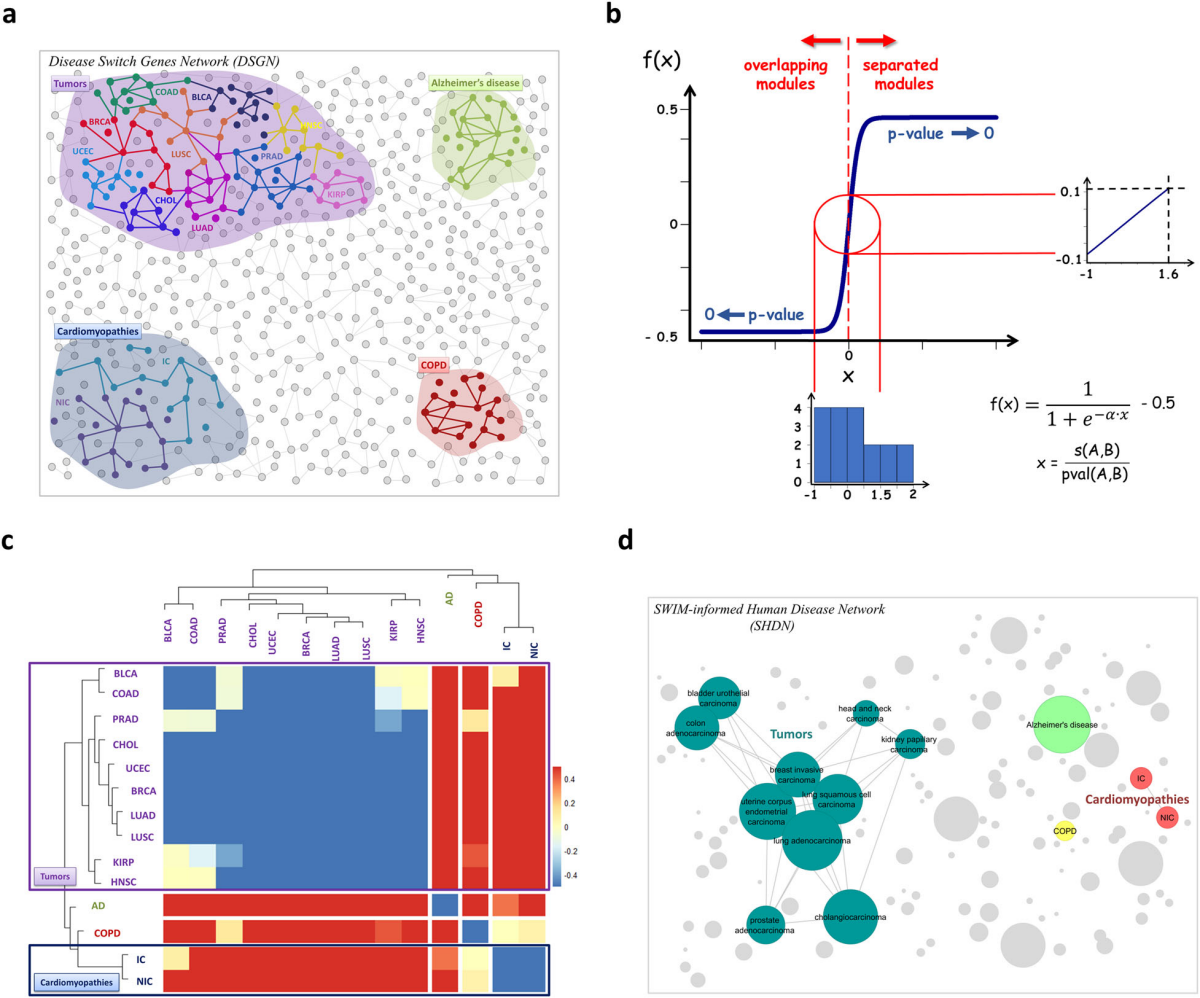
Results of the analysis. **a** Disease Switch Gene Network (DSGN). Schematic representation of disease modules informed by switch genes in the human interactome. Switch gene products (switch proteins) were colored based on the disorder class to which they belong. Gray nodes and the corresponding links are theoretical and represent non-switch proteins hypothetically connected to switch proteins within the interactome. **b** Generalized measure of the module separation. The function *f*(*x*) is the generalized version of module separation defined in Eq. (3) (cf “Methods”). This function approaches its maximum value when the disease modules are significantly well-separated (*p*-value < 0.05), whereas it approaches its minimum value when the disease modules significantly overlap (*p*-value < 0.05). The value of *α* = 0.3 was chosen to have *f*(*x*) close to zero for statistically insignificant *p*-values (i.e., *f*(*x*) in [−0.1, 0.1] as highlighted by the red circle). The blue bars represent the frequency of *x* values, ranging from −1 and 1.6, Supplementary Data for statistically insignificant *p*-values, ranging from 0.09 and 1 (Supplementary Data 4). **c** SWIM-based disease dendrogram and symmetrical heatmap. The diseases modules identified by the disease-specific switch proteins in the human interactome are clustered by a complete linkage hierarchical clustering algorithm and by using the separation metric as a distance metric. Heatmap colors refer to the generalized separation metric, increasing from blue to red: shades of blue refer to *cognate disease modules* (i.e., *s* <0, *p*-value < 0.05); shades of red refer to *non-cognate disease modules* (i.e., *s* > 0, *p*-value < 0.05); and shades of yellow refer to *uncertain disease modules* (i.e., *p*-value > 0.05). **d** SWIM-informed Human Disease Network (SHDN). In the SHDN, each node corresponds to a distinct disorder, colored based on the disorder class to which it belongs. Labeled nodes correspond to the 14 diseases analyzed in this study, while unlabeled nodes are artificial and represent other diseases or developmental endotypes to be investigated. The size of each node is proportional to the number of switch genes involved in the corresponding disorder. A link between two diseases occurs if they share a substantial number of switch genes. AD Alzheimer’s disease, BLCA bladder urothelial carcinoma, BRCA invasive breast carcinoma, CHOL cholangiocarcinoma, COAD colon adenocarcinoma, COPD chronic obstructive pulmonary disease, HNSC head and neck squamous cell carcinoma, IC ischemic cardiomyopathies, KIRP kidney renal papillary cell carcinoma, LUAD lung adenocarcinoma, LUSC lung squamous cell carcinoma, NIC non-ischemic cardiomyopathies, PRAD prostate adenocarcinoma, UCEC uterine corpus endometrial carcinoma.

In particular, we observed that diseases displaying a pathobiological similarity (such as cancers or cardiomyopathies) shared a substantial number of switch genes reflected by overlapping disease modules (cognate modules), whereas diseases characterized by different pathological phenotypes (such as inflammatory lung diseases and AD) showed specific switch genes reflected in non-overlapping disease modules (non-cognate modules). These two situations are presented in Fig. 3a, where the projection of disease-specific switch gene products (disease-specific switch proteins) on the PPI network, denoted the Disease Switch Gene Network (DSGN), is represented. In the DSGN, disease-specific switch proteins with their corresponding interactions are colored based on the disorder class to which they belong, and all cognate modules (sharing a substantial number of disease-specific switch proteins) are represented as one coalesced module colored with a less intense color corresponding to the disease class. It is worth noting that the specificity of the DSGN is twofold: it is constructed starting from genes (1) that are predicted to have a key role in transcriptional rewiring (co-expression analysis) for a tissue-specific experiment (the “specific” side of the DSGN), and (2) that are related by interactions on the PPI network that are identified using various techniques under different specific experimental and biological conditions (the “universal” part of the DSGN).

### Functional enrichment analysis of overlapping SWIM-informed disease modules

DSGN provides an intuitive visualization of the phenotypic relatedness among diseases, clearly showing how, for example, the broad tumor disease module, encompassing several overlapping cancer-related modules, is well-separated from the COPD module, as well as from the cardiomyopathies disease module and the AD module (Fig. 3a). To provide a biological interpretation of these findings, we extracted the overlapping switch genes within the disease modules related to tumor and cardiomyopathies classes, and we performed a functional enrichment analysis by querying both KEGG pathways^38^ and Gene Ontology (GO)^39^ databases.

Among the tumor class, we found a prevalent set of 26 switch genes recurring across multiple tumors that were all overexpressed in tumor tissues (Supplementary Data 2) and appeared primarily involved in the regulation of cell cycle, which is a fundamental and tightly controlled process under physiological circumstances. Specifically, these tumor-recurring switch genes appeared functionally enriched (adjusted *p*-value < 0.05) in the cell cycle and progesterone-mediated oocyte maturation KEGG pathways, and include cyclin A2 (CCNA2), cyclin B2 (CCNB2), and polo-like kinase 1 (PLK1); as well as in the G2/M phase transition and the mitotic spindle checkpoint GO biological processes, including the forkhead transcription factor (FOXM1), MYB proto-oncogene like 2 (MYBL2), the NIMA related kinase 2 (NEK2), the BUB1 mitotic checkpoint serine/threonine kinase B (BUB1B), Aurora B kinase (AURKB), centromere protein F (CENPF), and the dual specificity protein kinase (TTK). Moreover, by evaluating the enrichment of known binding motifs in their promoter regions, this set of 26 tumor-recurring switch genes appeared to be putatively co-regulated by the nuclear transcription factor Y (NF-Y) family (NF-YA, NF-YB) and the E2F transcription factor family (E2F4/E2F6), known to participate in the regulation of progression through the cell cycle.

By contrast, we found a set of 29 switch genes shared between the two cardiomyopathies that were all downregulated in the disease (Supplementary Data 2) and appeared functionally enriched (adjusted *p*-value < 0.05) in the cardiac muscle contraction KEGG pathway, including cardiac-type troponin T2 (TNNT2), myosin light chain 3 (MYL3), the subunits 5A and 7B of the cytochrome c oxidase (COX5A and COX7B), the ubiquinol-cytochrome c reductase core protein 1 (UQCRC1), and cytochrome c1 (CYC1).

### SWIM-based estimation of disease relationships

In order to distinguish better among the three topologically different situations (cognate, non-cognate, and modules of uncertain overlap), we combined the module separation defined in Eq. (1) and its statistical significance (*p*-value) into a *generalized measure of module separation* defined in Eq. (3). This generalized version $\tilde s$ of module separation (Fig. 3b) was used to elucidate better the relationships among diseases. In fact, we performed a hierarchical clustering of the 14 analyzed diseases using the module separation measure as a distance metric, with the generalized $\tilde s$ coded by color scale as illustrated in the associated heatmap (Fig. 3c and Supplementary Data 4). Shades of yellow refer to poorly informative interactions between diseases, corresponding to modules of uncertain overlap in the interactome; shades of blue associate with strictly linked diseases, corresponding to overlapping modules in the interactome (cognate modules); and shades of red quantify the distance between diseases whose corresponding modules are distant from each other in the interactome (non-cognate modules). We found two main clusters: one including all tumor datasets (violet in Fig. 3c), and one including the two cardiomyopathies (dark blue in Fig. 3c) along with AD and COPD datasets as isolated branches, showing a direct relation between the pathobiological similarity of diseases and their relative distance in the human interactome.

### Building the SHDN

Using the generalized separation measure, the SHDN was built, where each node corresponds to a distinct disorder and the occurrence of a link between two disorders depends on the extent to which their corresponding modules are in the immediate vicinity of each other (Fig. 3d). The underlying hypothesis is that disease modules closer to each other than to other network components are more likely to share common switch genes and etiology mechanisms.

To correct the problem of not having a fully connected network, we define a threshold on the values the generalized separation measure can assume that directly reflects the existence or not of a link between two nodes. Thus, given a module, the generalized separation measure with another module must be less than the 75th percentile of the distribution of the negative values of the generalized separation measure between the given module and all others in order to produce a link between the two corresponding diseases. Nodes in this network are colored based on the disorder class to which they belong.

### Comparison between SWIM-informed and interactome-based disease modules

**Fig. 4:**
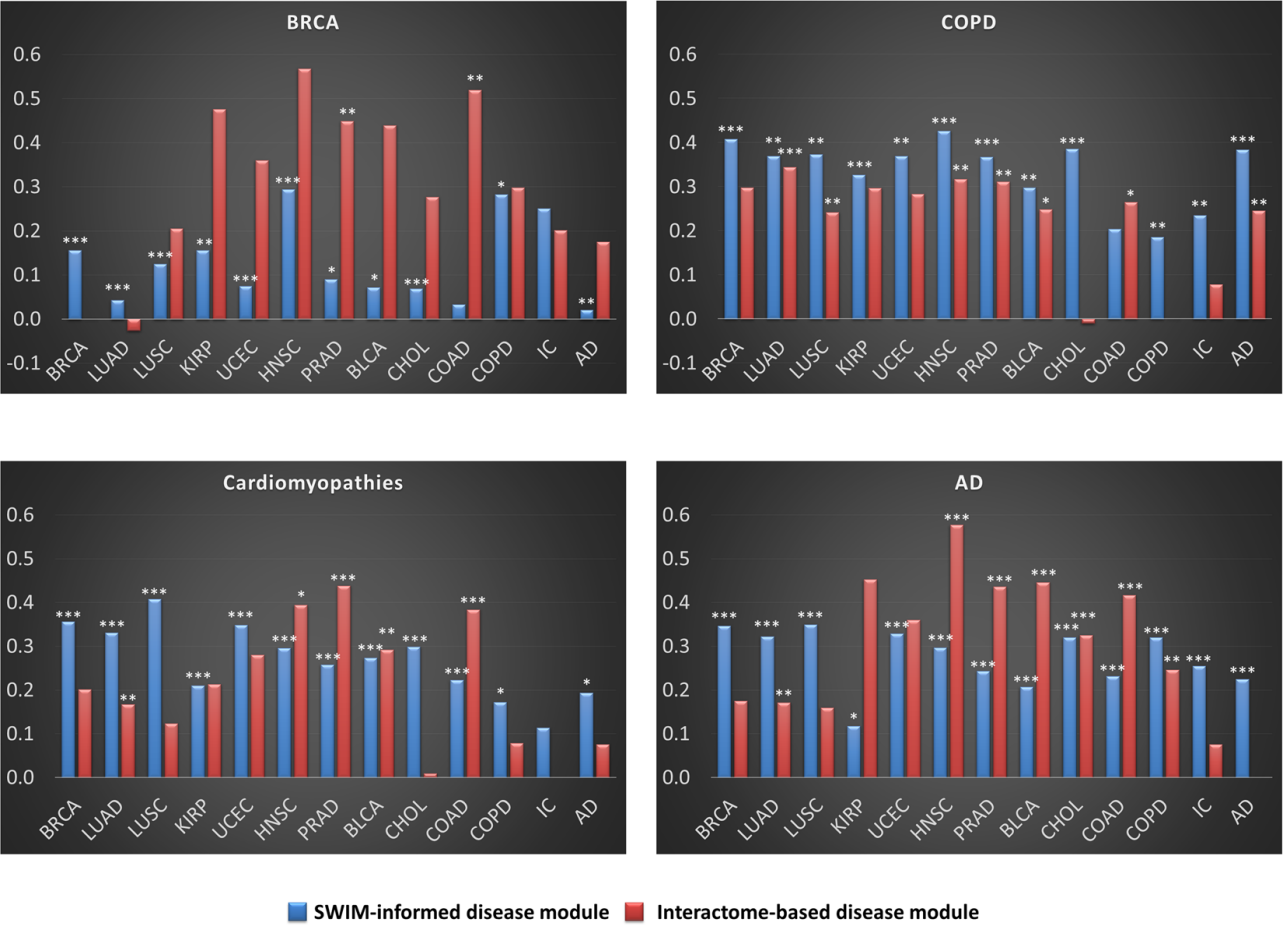
Comparison between SWIM-informed and interactome-based disease modules. The plots show the values of the non-Euclidean separation distance (Eq. 1, cf. “Methods”) computed between the interactome-based disease module corresponding to the disease reported in the title and SWIM-informed (blue bars) or interactome-based (red bars) disease modules, for all the diseases included in this study. We applied a degree-preserving randomization procedure to assess the statistical significance of each separation value, and we calculated all *p*-values by applying a two-tailed *z* test. The stars flag levels of significance for three of the most commonly used levels: *p*-value < 0.05 is flagged with one star (*); *p*-value < 0.01 is flagged with two stars (**); and *p*-value < 0.001 is flagged with three stars (***).

In order to analyze how the SWIM-informed disease modules (i.e., nodes in SHDN) are related to the interactome-based disease modules derived from disease genes in databases, we computed the non-Euclidean separation distance (Eq. 1) between the SWIM-informed disease modules and the interactome-based disease modules for each disease included in this study. We first retrieved the lists of disease genes from the DisGeNET database^40^, then built interactome-based disease modules and compared their distance from the modules in SHDN. We observed that SWIM-informed disease modules do not overlap with the interactome-based disease modules for the same disorder class (Fig. 4 and Supplementary Data 5), whereas they do among themselves. It is worth noting that although SWIM-informed disease modules do not overlap with interactome-based disease modules for the diseases analyzed, they do have lower separation values than those observed among interactome-based disease modules themselves. The lack of overlap between SWIM modules and interactome-based disease modules may reflect the partial knowledge of the number of genes associated with various diseases as well as the current incompleteness of the human interactome. An alternative explanation may lie in the differences in scale and influence across the interactome. The SWIM modules, by virtue of their basis in correlation among switch genes whose expression could be regulated by common transcription factors, reflect actions that may also occur “at-a-distance” in the interactome (i.e., long-range interactions), likely reflecting concomitant modulation of functionally distinct and separate submodules that inform phenotype. By contrast, the interactome-based disease modules are, instead, strictly defined on the basis of physical interactions among disease proteins in proximity to one another a local neighborhood of the PPI (i.e., short-range interactions), without reflecting the longer-range influences of hierarchical regulatory features. With regard to diseases characterized by different pathological phenotypes (such as COPD vs Alzheimer’s or Cardiomyopathies), the separations between SWIM modules and interactome-based disease modules or among interactome-based disease modules are similar.

## Discussion

The present study allowed us to demonstrate the relevance of switch genes in the context of network medicine and, in particular, their relation to the definition of disease genes. As broadly established^1,2^, disease genes have unique, quantifiable characteristics that distinguish them from other genes. From a network perspective, this observation translates into the verification that disease genes do not map randomly to the interactome, but, rather, manifest detectable correlations between their location and their network topology. This observation has led to a series of widely used hypotheses and organizing principles that tie the interactome to human diseases. These are summarized as follows: (i) the *local hypothesis*, according to which proteins involved in the same disease have an increased tendency to interact with each other; (ii) the *disease module hypothesis*, according to which proteins involved in the same disease show a tendency to cluster in connected subnetworks (or connected components), within which one of them is often much larger than the others (LCC); (iii) the *functional coherence hypothesis*, according to which genes in a disease module are often involved in the same biological process(es); and (iv) the *shared components hypothesis*, according to which related diseases are located in the same interactome neighborhood from which unrelated diseases are separated. We will next discuss how the results presented in this study support the validity and applications of those hypotheses with respect to switch genes.

*Local hypothesis.* Our work over the last decade demonstrated that switch genes appear consistently co-expressed in each disease studied thus far, showing coherent patterns of correlation that could presuppose possible co-regulation. Here, we have systematically demonstrated that these co-expression patterns turned into disease-specific subgraphs when mapped to the PPI network, whose nodes show a higher tendency to interact with each other more frequently than expected by chance (Fig. 2 and Supplementary Data 3). This observation confirms a fundamental hypothesis of interactome-based approaches to human disease, the *local hypothesis*, that genes associated with the same disease are not scattered randomly in the interactome, but aggregate in local, disease-specific neighborhoods.

*Disease module hypothesis*. By exploring the structural and topological properties of these disease-specific neighborhoods, we observed that they were composed of a dominant connected component, viz., to the LCC, whose size is significantly greater than expected by chance (Fig. 2 and Supplementary Data 3). This dominant component constitutes a highly connected and locally dense subgraph of the interactome, as witnessed by the number of its interactions, which are greater than expected by chance (Fig. 2 and Supplementary Data 3). We conclude that the LCC of each disease-specific subnetwork, built starting from switch genes, corresponds to their specific disease modules, fulfilling the *disease module hypothesis*.

*Functional coherence hypothesis*. We next extracted the switch genes within the disease modules, and, performed a functional enrichment analysis by querying both KEGG pathways^38^ and GO^39^ databases. Among the tumor class, we found a prevalent set of 26 switch genes recurring across multiple tumors that were all overexpressed in tumor tissues and appeared primary involved in the regulation of cell cycle.

For cardiomyopathies, we found a prevalent set of 29 switch genes shared between the two cardiomyopathies that were all downregulated in the disease and appeared functionally enriched in the cardiac muscle contraction KEGG pathway. Among them, we found TNNT2, a tropomyosin-binding subunit of the troponin complex that is located on the thin filament of striated muscles and regulates muscle contraction in response to alterations in intracellular calcium ion concentration; MYL3, referred to also as the ventricular isoform, whose mutations have been identified as a cause of mid-left ventricular chamber-type hypertrophic cardiomyopathy; and the ubiquinol-cytochrome c oxidoreductase complex that is part of the mitochondrial electron transport chain, which drives oxidative phosphorylation, playing an important role in the mitochondrial respiratory chain. This observation confirms that disease-specific switch genes fulfill the *functional coherence hypothesis*, being involved in closely disease-related cellular functions.

*Shared components hypothesis*. We have shown that switch genes may also belong to several disease modules, implying that disease modules may overlap, and, thus, perturbations in one disease module can disrupt pathways of other interlinked disease modules, as well. By building the SHDN, in which nodes are diseases and a link occurs if they share a substantial number of common switch genes, we quantified and visualized the overlap between the disease-associated switch gene modules (Fig. 3d). Although the SHDN was generated independent of any a priori knowledge of disease category, the resulting network is visibly clustered according to major disease classes, where cancers and cardiomyopathies represent the most connected disease classes, in contrast to COPD and AD, which appear as individual disorders. Clustering of nodes of similar color (denoting the same disease class) reflected the fact that similar pathophenotypes have a higher likelihood of sharing genes than do pathophenotypes that belong to different disease classes (Fig. 3d). For example, cancers formed a tightly interconnected and easily detectable cluster, which was held together by a small group of genes that were associated with multiple cancers. Therefore, the SHDN clearly shows how network modules identified by switch genes are highly specific for each disease category and tend to group according to similar pathobiological phenotypes, implying that disease-associated switch gene modules fulfill the *shared components hypothesis*.

The set of 26 tumor-recurring switch genes across multiple tumors showed a marked functional annotation enrichment in cell-cycle-related terms, specifically regulation of the G2-to-M transition. Among them, we found FOXM1, which is a transcription factor with a crucial, central role in cancer development^41^. Indeed, FOXM1 overexpression was detected in a variety of human cancers and is associated with poor clinical prognosis^42,43^; it drives the expression of critical genes involved in the regulation of different cancer hallmarks including high proliferation, invasion, drug resistance, and angiogenesis. In particular, a very recent study demonstrated that FOXM1 physically interacts with the architectural transcription factor HMGA1 to promote tumor angiogenesis cooperatively both in vitro and in vivo models^44^.

Interestingly, among the positive nearest neighbors of FOXM1 in the GENs identified by SWIM methodology, we found both HMGA1 and several well-known pro-angiogenic factors^45,46^ such as tumor necrosis factors (TNFs), fibroblast growth factor (FGF), the matrix metalloproteinases (MMPs), together with other genes involved in the regulation of angiogenesis such as ADM2, ESM1, E2F7, E2F8, and E2F2. Yet, the negative nearest neighbors of FOXM1 included genes functionally related to metabolic process, as a well appreciated mark of tumor transformation^47^.

This set of 26 tumor-recurrent switch genes appeared coregulated by two major transcription factors (viz., E2F and NF-Y), already known to play key roles in cell cycle regulation and transformation. In particular, the role of NF-Y in controlling cell proliferation has been widely established based on the following findings^48–51^: it controls the expression of several key regulators of the cell cycle; NF-Y silencing impairs G2/M progression and induces apoptosis; widespread activation of G2/M and anti-apoptotic genes requires NF-Y; NF-Y and mutant p53 physically interact, upregulating the expression of many cell-cycle-related genes in response to DNA damage; and NF-Y overexpression increases cell proliferation. Yet, E2F4 may function as an activator of genes implicated in positive regulation of the cell cycle, including MYBL2 (ref. ^52^), whose overexpression in transgenic mice leads to the development of tumors, and mutated E2F4, which has been reported in various human tumors, providing evidence for its oncogenic activity^53–55^.

Taken together, these findings suggest a model wherein NF-Y, in collaboration with E2F4 and/or MYBL2 complex, binds to and activates transcription of E2F/NF-Y-dependent switch genes accelerating the late phase of the cell cycle by promoting angiogenesis with a consequent increase of cancer progression, together with a rewiring of some metabolic pathways, hallmarks of the malignant transformation.

These results support the hypothesis that correlation-based network analysis may move toward causation highlighting functionally coordinated genes whose common perturbations in expression pattern and abundance may contribute to the pathobiological phenotype. In addition, this approach may aid in the identification of biologically significant PPIs (e.g., HMGA1–FOXM1 interaction) of the human interactome, which remains incomplete at the current time.

By definition, disease genes refer to genes with mutations that are known to have a phenotypic impact, e.g., sequence alterations that are causal for Mendelian diseases or variants that increase the susceptibility to complex diseases or cancers^2,40,56–58^. However, fundamental insights toward the discovery of disease biomarkers can also stem from measuring transcript abundance or gene expression patterns for given phenotypes (case-control) across multiple samples, whose changes could reveal tissue/cell-specific co-expression relationships in the context of the disease^2,3^.

Here, we demonstrated that switch genes simultaneously satisfy all of the widely used hypotheses and organizing principles formalized by the network medicine construct that tie the interactome to human diseases, in the same way as disease genes themselves do. Thus, the identification of switch genes could allow the systematic prediction of novel disease–gene associations whose perturbations in their expression pattern and abundance contribute to the pathobiological phenotype, as well.

Being context-specific by definition, switch genes can be used to integrate the human interactome with the cell-type or tissue-specific manifestations that characterize many diseases. The driving principle is to use tissue-specific expression information arising from switch genes to filter the global interactome for interactions that are feasible in a given tissue (i.e., both switch interaction partners are present). Furthermore, SWIM methodology may even help in the identification of biologically significant, yet unmapped, PPIs connecting proteins (i.e., switch gene products) on the basis of their co-expression profiles and network-based proximity. In this sense, SWIM supports the link prediction process aiding in increasing the coverage of the human interactome, which is incomplete at the current time^6^.

Taken together, these observations make the accurate identification of switch genes an important step toward a systematic understanding of the networked nature of human pathobiology. We believe that the SWIM-informed approach to protein interaction networks presented here, if broadly applied, would significantly catalyze innovation in the discovery of the determinants of human diseases.

## Methods

### SWIM software

In order to identify disease-specific switch genes, we exploited the SWIM software, a program for gene co-expression network mining developed in MATLAB with a user-friendly Graphical User Interphase (GUI) and freely downloadable^19^.

### Consolidated human interactome

To build the comprehensive human interactome, we compiled human physical molecular interaction data from different sources, including PPIs, protein complexes, kinase–substrate interactions, and signaling pathways. PPIs from several high-throughput yeast-two-hybrid studies as well as high-quality PPIs from the literature were compiled from the CCSB Human Interactome^59–63^. We also collected binary PPIs from other laboratories^64,65^. A protein complex is a group of two or more associated polypeptide chains linked by non-covalent associations. Protein–protein co-complex interactions were compiled from different high-profile publications^66–72^. In addition, we also incorporated experimental signaling interactions and kinase–substrate interactions, as well as high-quality literature-based signaling interactions involved in various biological pathways^73–76^. This new version of the consolidated human interactome has 16,470 proteins and 233,957 interactions after incorporating the latest reference map of the human binary protein interactome^77^.

### Ischemic and non-ischemic cardiomyopathy dataset

This dataset is available through the GEO public repository at accession number GSE76293 published on February 10, 2014 (ref. ^78^). Data include a complete RNA-sequencing transcriptome profiling from left ventricular apex tissue from human failing hearts and from non-failing control hearts, with a total of 40 samples: 16 ischemic subjects, 16 non-ischemic subjects, and 8 control heart subjects. High-throughput RNA-sequencing data correspond to normalized expression data created using the reads per kilobase of transcript per million mapped reads (RPKM) procedure to perform the normalization. By running SWIM on ischemic (or non-ischemic) subjects with respect to controls samples, we extracted a list of 81 switch genes, mapped to 68 proteins in the human interactome (less than the total number of switch genes owing to the incompleteness of the interactome).

### Alzheimer’s disease dataset

This dataset is available through the GEO public repository at accession numbers GSE63060 (batch 1) and GSE63061 (batch 2) published on August 05, 2015 (ref. ^79^). Data include expression profiling by array related to AD and control samples (CTL) originating from the EU funded AddNeuroMed Cohort^80^, which is a large cross-European AD biomarker study relying on human blood as the source of RNA. In particular, batch 1 (GSE63060) comes from array A-MEXP-1171-Illumina HumanHT-12 v3.0 Expression BeadChipm and has a total of 249 samples (145 AD, 104 CTL); whereas batch 2 (GSE63061) comes from array A-GEOD-10558-Illumina HumanHT-12 V4.0 expression beadchip and has a total of 273 samples (139 AD and 134 CTL). The probe-sets were mapped to official gene symbols using the relative platform (GPL6947-13512 for GSE63060 and GPL10558-50081 for GSE63061) available from the GEO repository. Multiple probe measurements of a given gene were collapsed into a single gene measurement by considering the mean. By matching genes based on gene symbols, we created a single merged dataset with both batches; we ran Combat function from R/Bioconductor package SVA to correct for batch-specific effects. Finally, we obtained a data matrix of 19,460 gene symbols (rows) and 522 samples (columns) including 284 AD and 238 CTL. By running SWIM on AD subjects with respect to controls samples, we extracted a list of 375 switch genes, mapped to 301 proteins in the human interactome.

### TCGA datasets

A selection of ten tumor types were recovered from the original study^19^, where a collection of tumor expression data from high-throughput RNA- and miRNA-sequencing were downloaded from the TCGA data portal on December 6, 2014. High-throughput RNA-sequencing data correspond to level 3 data (i.e., normalized expression data) from RNASeq Version 2 created using MapSplice to do the alignment and RSEM to perform the quantification and normalization. MiRNA-sequencing data correspond to level 3 data (i.e., normalized expression data) created using the RPKM procedure to perform the normalization. In the original study^19^, only cancer datasets including at least seven patients with tumor and matched-normal samples (i.e., the matched-normal tissue is defined as the tissue that is adjacent to the tumor and taken from the same patient) for both RNA- and miRNA-sequencing experiments were retained for subsequent analysis. Our selection of ten tumor types, detailed in Table 1, corresponds to tumor types whose switch genes formed a statistically significant module in the human interactome (i.e., showing statistically significant *module significance* for all the three measurements: size of LCC, edges of LCC, and total number of interactions), and showed a statistically significant (*p*-value < 0.05) *module separation* measure in at least 70% of the comparisons.

**Table 1:** Summary of TCGA datasets.

Acronym	Tumor name	No. of samples	No. of switch genes	No. of proteins in human interactome
BLCA	Bladder urothelial carcinoma	38 (19 matched-normal)	297	203
BRCA	Breast invasive carcinoma	206 (103 matched-normal)	257	223
CHOL	Cholangiocarcinoma	18 (9 matched-normal)	324	285
COAD	Colon adenocarcinoma	52 (26 matched-normal)	264	217
HNSC	Head and neck squamous cell carcinoma	82 (41 matched-normal)	109	96
LUAD	Lung adenocarcinoma	36 (18 matched-normal)	366	321
LUSC	Lung squamous cell carcinoma	76 (38 matched-normal)	274	254
KIRP	Kidney renal papillary cell carcinoma	46 (23 matched-normal)	133	119
PRAD	Prostate adenocarcinoma	104 (52 matched-normal)	229	177
UCEC	Uterine corpus endometrial carcinoma	14 (7 matched-normal)	395	297

### COPD dataset

Data for COPD were recovered from the original study^22^ in which the SWIM software was applied to the COPD dataset. The dataset is available through the GEO public repository at accession number GSE76925 published on March 29, 2017 (ref. ^81^). Data include microarray gene expression profiling of lung or airway tissue from subjects with COPD obtained using HumanHT-12 BeadChips (Illumina, San Diego, CA). A total of 111 COPD cases and 40 control smokers with normal lung function were collected; all subjects were ex-smokers. The probe-sets were mapped to official gene symbols using the platform GPL10558 (Illumina HumanHT-12 V4.0 expression beadchip) available from the GEO repository. Multiple probe measurements of a given gene were collapsed into a single gene measurement by considering the mean. A list of 61 switch genes was extracted, mapped to 55 proteins in the human interactome.

### Module separation

To evaluate disease–disease relationships, we computed the non-Euclidean separation distance, which measures the disease modules’ overlap^6^, as follows:

1 $$s\left( {A,B} \right) = p_{AB} - \frac{{p_{AA} + p_{BB}}}{2}$$

where, *p*(*A, B*) is the module proximity:

2 $$p\left( {A,B} \right) = \frac{1}{{\left| A \right| + \left| B \right|}}\left[ {\mathop {\sum}\nolimits_{a{\it{\epsilon }}A} {\mathop {{{\mathrm{min}}}}\limits_{b{\it{\epsilon }}B} d\left( {a,b} \right)} + \mathop {\sum}\nolimits_{b{\it{\epsilon }}B} {\mathop {{{\mathrm{min}}}}\limits_{a{\it{\epsilon }}A} d\left( {b,a} \right)} } \right]$$

and *d*(*a*, *b*) is the shortest distance between switch gene *a* of module *A* and switch gene *b* of module *B*. A positive value for the separation measure indicates that the two lists of switch genes mapped to proteins are topologically well-separated in the human interactome, whereas a negative value for the separation measure indicates that two switch gene sets are located in the same network neighborhood and, thus, form overlapping modules with some switch genes belonging to the two disease modules simultaneously.

To evaluate the significance of the network separation parameter across two disease-specific modules (A, B) of switch genes, we built a reference distance distribution corresponding to the expected distance between two randomly selected groups of proteins of the same size and degree distribution as the original two sets of switch genes in the human interactome. The random selection was repeated 1000 times in order to build the reference distance distribution. The module separation measure across the two lists of switch genes was *z*-score-normalized by using the mean and the standard deviation of the reference distribution. Subsequently, the *p*-value for the given *z* statistic was calculated. A *p*-value < 0.05 indicates that the module separation in the human interactome of the two lists of switch genes is more (or less, see below) than expected by chance.

### Generalized measure of module separation

The module separation defined in Eq. (1) and its statistical significance (*p*-value) were combined into a *generalized measure of module separation* modeled as the following sigmoidal function:

3 $$\tilde s\left( {A,B} \right) = \frac{1}{{1 + e^{ - \alpha \cdot \frac{{s\left( {A,B} \right)}}{{pval\left( {A,B} \right)}}}}} - 0.5$$

where *s*(*A*, *B*) is the module separation, *pval*(*A*, *B*) is the corresponding *p*-value, and *α* is a smoothing parameter: the greater the *α*, the steeper the function (Fig. 3a).

This generalization of *s*(*A*, *B*) is a bounded function returning a value that monotonically increases from −0.5 to 0.5, and explicitly considers the statistical significance (i.e., the *p*-value) of the observed module separation between each disease pair (*A*, *B*): the lower the *p*-value, the greater the absolute value of $\tilde s(A,B)$ with $\left| {\tilde s} \right|$ approaching 0.5 as the *p*-value approaches 0. In particular, we chose a quite small value of *α* (=0.3) in order to emphasize better the differences between negative (overlapping disease modules) and positive (well-separated disease modules) values of *s* when the corresponding *p*-value is small and statistically significant (i.e., *p*-value < 0.05). Note that for statistically insignificant *p*-values (i.e., *p*-value > 0.05), the $\tilde s(A,B)$ clearly shows its exponential behavior near zero (Fig. 3a).

### Functional and motif enrichment analysis

The functional enrichment analysis was performed using EnrichR web tool^82^. Binding motif enrichment analysis in promoter regions (identified as genomic regions spanning from −450 to +50 nucleotides with respect to transcription start sites) was performed using Pscan^83^, which employs the JASPAR 2018 motif collection^84^. *p*-Values were adjusted with the Benjamini–Hochberg method, and a threshold equal to 0.05 was set to identify functional annotations and regulatory motifs significantly enriched among the selected switch gene lists.

## Supplementary information

Supplementary Data captions

Supplementary Data 1

Supplementary Data 2

Supplementary Data 3

Supplementary Data 4

Supplementary Data 5

Reporting-summary

## Data Availability

The accession codes, unique identifiers, or web links for publicly available datasets are provided in Datasets subsection of “Methods”. Data associated to figures are provided as supplementary material.

## Code availability

SWIM code is freely available at www.nature.com/articles/srep44797 (Supplementary Information).
