# Supplementary material for: Gene co-expression in the interactome: moving from correlation toward causation via an integrated approach to disease module discovery
Source: NPJ Syst Biol Appl. 2021 Jan 21;7:3. doi: 10.1038/s41540-020-00168-0 (PMC7819998; doi:10.1038/s41540-020-00168-0)
Supplement: Supplementary file 1 — Supplementary Data captions [file 41540_2020_168_MOESM1_ESM.docx]

# Supplementary Data description

**Supplementary Data 1. Disease-specific Gene Expression Networks (GENs).** This table reports the gene expression networks for each analyzed disease on separate sheets.

**Supplementary Data 2. Switch genes.** This table reports the lists of switch genes along with their statistics for each analyzed dataset on separated sheets.

**Supplementary Data 3. SWIM-informed disease modules.** This table reports the size of the largest connected component (LCC), the number of edges in the LCC, and the number of total interactions of SWIM-informed disease modules included in this study, together with the corresponding p-values.

**Supplementary Data 4. SWIM-informed disease modules separation.** This table reports: [top] the matrix of separation values computed between each pair of SWIM-informed disease modules included in this study and [bottom] the matrix of their corresponding p-values.

**Supplementary Data 5. Comparison between SWIM-informed and interactome-based disease modules.** This table is composed of two separated sheets. The first sheet reports: [top] the matrix of separation values and of their corresponding p-values computed between SWIM-informed and interactome-based disease modules for all diseases included in this study; [bottom] the matrix of separation values and of their corresponding p-values computed between the interactome-based disease modules themselves. The second sheet contains the bar plots presented in Figure 4.
